# Enhanced cancer cell sorting using lab-on-a-disk pattern design with magnetic and centrifugal forces

**DOI:** 10.3389/fbioe.2025.1611313

**Published:** 2025-08-01

**Authors:** Bill Cheng, Wei-Cheng Chao, Yi-Han Chen, Yao-Tsung Lin

**Affiliations:** ^1^ Graduate Institute of Biomedical Engineering, National Chung-Hsing University, Taichung, Taiwan; ^2^ Doctoral Program in Tissue Engineering and Regenerative Medicine, National Chung-Hsing University, Taichung, Taiwan; ^3^ Graduate Institute of Precision Manufacturing, National Chin-Yi University of Technology, Taichung, Taiwan

**Keywords:** microfluidic flow, cell sorting, non-invasive method, lab-on-a-disk, rapid detection

## Abstract

Using microfluidic flow for biological detection is a non-invasive method that can replace traditional invasive testing methods to achieve fast and accurate results. The design of the detection device and lab-on-a-disk (LoaD) can impact performance in accurately identifying biological features. Therefore, we created a novel device to extract cancer cells from a heterogeneous cell population by centrifugal-force-driven microfluidic flow and magnetic labeling. Two-stage centrifugal force and a specially designed LoaD were used to drive microfluidic flow and control its movement to designated areas. The purpose was to allow the CD44 antibody–magnetic bead complex (CD44 beads), which specifically binds to the abundantly present CD44 receptors on identifiable cancer cells, to flow into the reservoir well, while the biological mixture containing the cancer cells is retained in the capture well. Fluorescence imaging as well as flow cytometric analysis revealed the successful retention of the microbead-bound cancer cells in the magnetic area, while the remaining biological mixture was retained in the reservoir area. The entire separation process took less than 2 h.

## 1 Introduction

Blood and saliva are classified as liquid biopsies and are commonly utilized in microfluidic applications. These fluids exhibit unique characteristics such as viscosity, surface tension, electrical conductivity, and pH, allowing small-volume samples to be used for the detection of viruses, diseases, and drug residues. Microfluidic systems are capable of isolating specific biological components from complex mixtures, making them effective alternatives to traditional invasive sampling techniques (Li et al., 2025; Lin et al., 2024; Petruzzellis et al., 2024). To address the growing demand for minimally invasive diagnostics, various lab-on-a-disk (LoaD) platforms—also referred to as “microfluidic chips”—have been developed using different actuation mechanisms that include gravitational, capillary, centrifugal, pressure, electrical, acoustic, and magnetic forces (Liu et al., 2023; Farahinia et al., 2023; Warkiani et al., 2015; Panesar and Neethirajan, 2016). These systems enable disease diagnostics, drug residue analysis, and biomarker identification through precisely controlled fluid flow.

Despite these advances, many existing devices for cell sorting or cancer cell detection remain limited by large system size, high cost, and complex operating procedures. In response to these limitations, recent research has focused on developing compact, low-cost platforms that support accurate detection with simplified operation. Among the available techniques, centrifugally and magnetically driven microfluids are particularly attractive due to their high integration capacity, ease of use, and independence from external power sources or pumps (Burger et al., 2012). As such, they have been extensively applied in immunoassays, clinical diagnostics, and pathogen detection (Guan et al., 2020; Shin et al., 2018; Zhu et al., 2018). Sivaramakrishnan et al. (2020) represent a cell sorting technology that can enable a distinct population of cells to be isolated from a heterogeneous cell population with no or minimal contamination from other cell types or a complex biological sample such as blood. Zhu et al. (2020a) describe cell separation technologies that prevent isolated cells from being contaminated by the surroundings, thus allowing them to be further cultured for subsequent analysis or cell therapy. This is particularly important in some of the latest immunotherapeutic products, where endogenous cells such as T lymphocytes and dendritic cells are isolated from patients in the purest form before being sub-cultured and processed for subsequent cell therapy. Likewise, for diagnosis, biological samples such as circulating tumor cells (Wei et al., 2021) and blood-borne microorganisms such as *Staphylococcus aureus* must be purified for identification (Zhang et al., 2023). Centrifugal microfluidic devices also allow integration with external components, such as signal sensors or heating elements, to further enhance their functional scope (Kim et al., 2016). Therefore, they have been widely researched in clinical disease diagnosis.

Samples could be pre-stored on an LoaD and depend on rotating speed to realize microfluid flow, mixing, or transfer by centrifugal device (Nguyen et al., 2019). Therefore, the material selection and the pattern design of the LoaD are very important when using the centrifugal device to sort cells (Kuan et al., 2018). Polymethyl methacrylate (PMMA) is biocompatible and bioinert (Zhang et al., 2018) and is primarily used for disposable LoaD. In addition, the microrunner’s geometry will affect movement and reservation of the microfluidic flow from one specific area to another by the centrifugal device. Therefore, Lin Y. T. et al. (2021) proposed a pattern capable of controlling microparticle movement and localization by the centrifugal device. However, they did not explore the microfluidic flow and performance of cell sorting. Lin (2011) investigated high-speed flow regulation but did not optimize sequential flow control. Furthermore, high-speed rotation may damage fragile biological structures, suggesting the need for optimized flow control at moderate rotational speeds.

To address these concerns, this study presents a new centrifugal microfluidic system designed to achieve stable sequential flow by integrating tailored LoaD patterns with dual-mode rotation control. The system is evaluated for its performance in cancer cell sorting through magnetic labeling. In addition to laboratory-based diagnostics, the proposed LoaD system holds strong potential for deployment in resource-limited or rural settings where access to advanced medical infrastructure is scarce. Its simple operation, minimal instrumentation, and low manufacturing cost make it ideal for point-of-care testing (POCT). By enabling rapid, low-cost cancer cell detection without the need for external pumps or complex analytical equipment, our platform offers a promising solution for decentralized diagnostics, especially in developing countries or underserved areas. This accessibility could greatly enhance early disease detection and timely intervention in populations with limited healthcare resources.

## 2 Research procedure and methodology

### 2.1 Principle of centrifugal and magnetic adsorption

#### 2.1.1 Centrifugal force drives the microfluidic flow

Shear stress leads to microfluidic flow in the runner of the LoaD. We represent by ∂u/∂y the relationship of viscosity coefficient and shear stress within the fluid, which can be expressed as Formula 1 if the fluid is in the state of Newtonian fluid.

$$\tau =\mu \frac{\partial u}{\partial y}$$ (1)

where τ express the shear stress, μ express the viscosity coefficient, u express the velocity of fluid, and *y* represents the position of a unit element of the fluid in the runner. μ will vary with the function of y. Therefore, the force of a fluid element has two types: pressure or viscosity when the fluid element is under the space of *δx*, *δy*, and *δz*. Viscosity must be accompanied by shear force to be generated according to Formula 1, so the difference of the shear force can be expressed as Formula 2 when the fluid flow is under the runner of the LoaD.

$$\mu \frac{\partial^{2}u}{\partial y^{2}}\delta x\delta y\delta z$$ (2)

where δxδyδz expresses the unit volume and $\mu \frac{\partial^{2}u}{\partial y^{2}}$ expresses the viscosity of the unit volume. The fluid will create a pressure difference when the fluid flows downstream from upstream due to the difference of shear force leads to the fluid flow. The pressure difference can be expressed as Formula 3:

$$p_{x+\delta x}\delta y\delta z=\left(p_{x}+\frac{\partial p}{\partial x}\delta x\right)\delta y\delta z$$

$$∆p=-\frac{\partial p}{\partial x}\delta x\delta y\delta z$$ (3)

In addition, the main factor distinguishing the compressible from the incompressible flow is pressure. Therefore, we define the formula of fluid density as Formula 4:

$$\rho =\rho_{0}+∆\rho$$ (4)

where *ρ*_*0*_ is the density before being incompressible and ∆ρ is the variation of the fluid density. We consider it incompressible flow when ∆ρ/ρ<<1. Most microfluids belong to incompressible flow.

Centrifugal force is an inertial force. It can make the rotating object or fluid move away from its center of rotation so that the fluid will slide due to the velocity or acceleration in the radial direction. Therefore, we can give Formulas 5 and 6:

$$\omega =\lim_{∆t\to 0}\frac{∆\theta }{∆t}=\frac{d\theta }{dt}$$ (5)

$$\alpha =\lim_{∆t\to 0}\frac{∆\omega }{∆t}=\frac{d\omega }{dt}$$ (6)

Regmi et al. (2022) and Mason (2014) represent the fluid moves with inertial force driven by pressure, viscosity, and gravity when the fluid moves along the flow path. Centrifugal force equation is as in Formula 7:

$$f_{c}=m\cdot a=m\times r\cdot \omega^{2}$$ (7)

where *f*_*c*_ is the inertial force, m is the quality of the particle, *a* is the acceleration of the microfluidics, *Δθ* is the angle of the movement, and *ω* is the angular velocity.

#### 2.1.2 Dean flow drives microfluidic mixing

The microfluid flow velocity in the tube’s center is faster than near the tube wall when microfluid flows along a circular tubular channel. The microfluid in the center of the tube will be pushed to the outside, and the microfluid near the tube wall will be squeezed back to the inside, thus creating two opposite vortices. This phenomenon is called Dean flow. Therefore, the microfluid will create mixed effects. Dean flow can be represented as Formula 8 (Li et al., 2024):

$$De=Re\delta^{1/2}=\frac{\rho uL}{\mu }\sqrt{\frac{L}{2R}}$$ (8)

where *Re* is the Reynolds number, *L/2R* is the curvature ratio, *ρ* is the fluid density, *µ* is the fluid viscosity, u is the mean velocity of the flowing fluids, L is the characteristic length of the rectangular channel cross-section, and R is the runner radius.

Microfluid flow is laminar in the runner of the LoaD (Hoshino et al., 2011). It belongs to incompressible flow or Newtonian fluids and is therefore according to the Navier–Stokes equation (Mei and Qian, 2022):

$$\rho \left[\frac{\partial u}{\partial t}+\left(u\cdot \nabla \right)u\right]=-\nabla p+\mu \nabla^{2}u+f_{c}$$ (9)

where *ρ* is the density of the fluid, *u* is the velocity of the flow, *p* is the pressure, *µ* is the viscosity, and $f_{c}$ is the inertial force.

$$Re=\frac{\rho ud}{\mu }$$ (10)

where Re is the Reynolds number and d is the diameter of the runner in the LoaD. Re < 2,300 is the laminar flow (Bertsche et al., 2019).

#### 2.1.3 Magnetic force

The principle of cell sorting involves two forces on the cell: centrifugal (*f*_*c*_) and magnetic force (*f*_*m*_). The formula for magnetic force is as Equation 11 (Oh et al., 2018):

$$f_{m}=\frac{V∆x}{2\mu_{0}}\nabla B^{2}$$ (11)

where V is the volume of the magnetic particle, ∆x is the effective magnetic volumetric susceptibility, $\mu_{0}$ is the magnetic permeability of the vacuum, and B is the magnetic field intensity. The magnetic particles will be adsorbed by the magnetism when the magnetic force (*f*_*m*_) is over the centrifugal force (*f*_*c*_).

### 2.2 The fabrication of the centrifugal device

**FIGURE 1:**
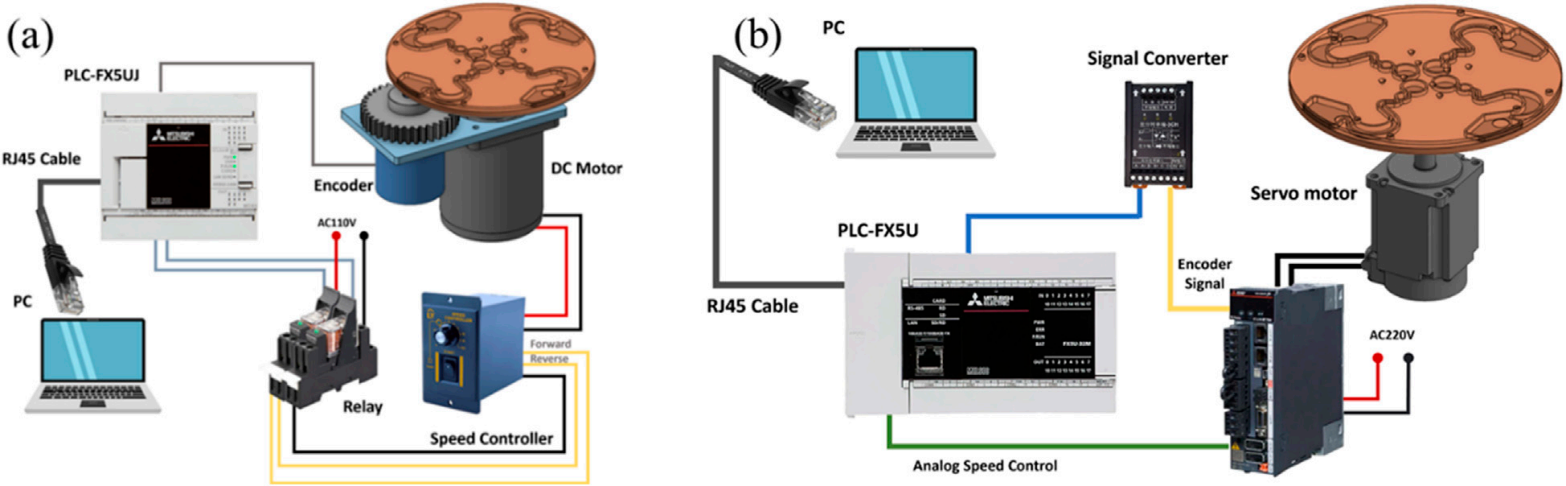
**(a)** DC motor system. **(b)** Servomotor system.

The novel centrifugal microfluidic detection device is created by the LoaD, magnet, motor, and control system. Easy operation, lower cost, and higher performance are our key points of novelty. However, it is a challenge to how to control the microfluidic steady flow in the runner of the LoaD (Lin, 2011; Acharya et al., 2023). The rotating device of the motor and the pattern of the LoaD are key points. Therefore, we use a brush motor and servomotor to compare the steady rotating issue for the LoaD. Figure 1 represents the system compositions for the brush motor and the servomotor; their specifications are illustrated in Table 1. The brush motor system components are the relay, speed controller, PLC, encoder, and computer. The servomotor system components are the servomotor, signal converter, speed controller, PLC, and computer.

**TABLE 1:** Components of the centrifugal controller system and specifications.

Components and specifications	DC motor system	Servo motor system
Motor device	Xajong DC Drive Motor 05SP-9032 Output 20 W DC 90 V, 0.34 A Max. 3,200 r.p.m Torque 0.75 Kg-cm	Mitsubishi AC Servo Motor HK-KT43W Output 400 W AC 108 V, 2.6A Max. 3,000 rpm/min, 0–250 Hz
Controller	Chang Yih NC-200 Input AC110∼220 V Output DC 90 V	Mitsubishi MR-J5-40A Power 400 W Input 3AC, 200–240 V, 2.6–4.5 A, 50/60 Hz Output 3 PH, 0–240 V, 0–590 Hz, 2.8A
Lab-on-a-Disc	Material: PMMA Dimension: Ø120 mm*t5.0 mm Mixing runner angle (θ): 45°, 50°, 55°, 60°, 65°, 70°, 75°, 80°, 85°, 90° Weight: 60 g Maximum volume of buffer well: 150 µL Maximum volume of sample well: 40 µL Maximum volume of reaction area: 250 µL	
Microfluidics	Coomassie Brilliant, Blue R-250, Max 120 µL	

**FIGURE 2:**
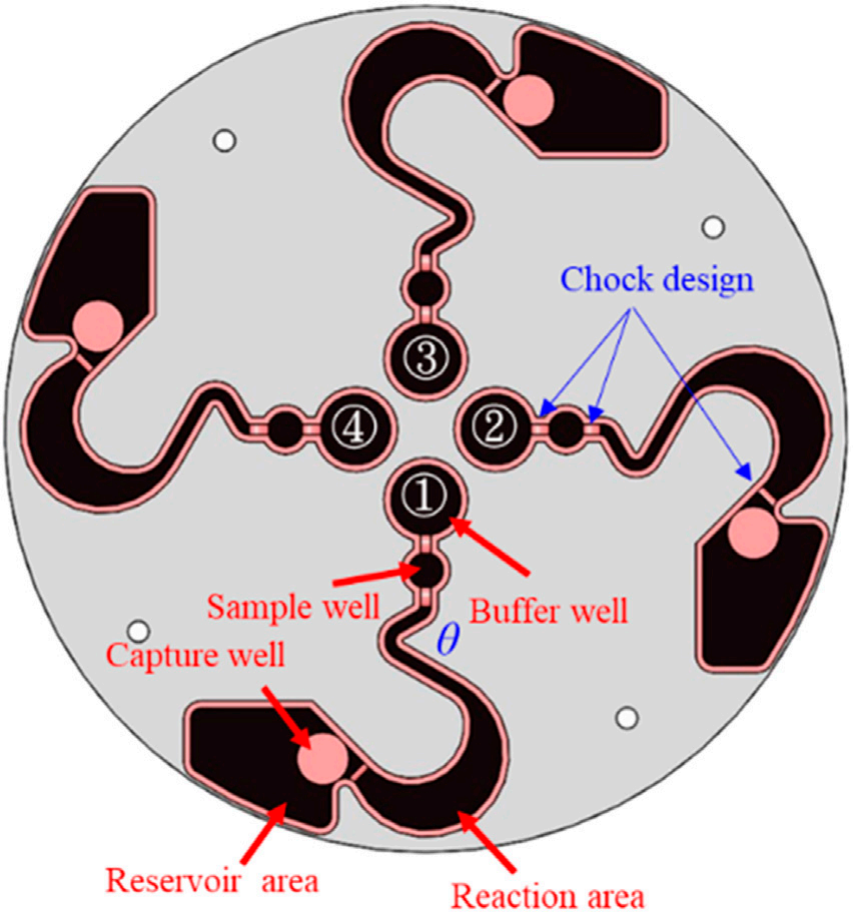
Pattern design of the LoaD (Lin Y. T. et al., 2021).

Lin Y. T. et al. (2021) used CFD software to design and simulate a novel LoaD. The detail specifications and pattern design of the LoaD are illustrated in Figure 2. The novel LoaD can control the micro-particles to flow, stay, and reflow in the runner of the LoaD by the different rotating mode. Therefore, we used the material of the PMMA to process the novel pattern of the LoaD by a CNC machine. The purpose was to test the performance of the microfluidic flow and control in this pattern of the LoaD. We used 95% EtOH and deionized water to wash the pattern of the LoaD and dry it after CNC processing. Then, we used cover and pressure-sensitive adhesive to combine the LoaD (Kuo et al., 2023; Zhang et al., 2018).

### 2.3 Cancer cell purification method

Human breast cancer cells MDA-MB-231 (ATCC, Cat# HTB-26) and human noncancer cells HEK293 (ATCC, Cat# CRL-1573) were cultured in a separate culture dish in DMEM-high glucose (Gibco, Cat# 12100046) with 10% fetal bovine serum (Gibco) and 1% penicillin–streptomycin (Gibco). The day before the experiment, MDA-MB-231 and HEK293 were stained with Mitotracker Red (Thermo Fisher Scientific, Cat# M7512) and Mitotracker Green (Thermo Fisher Scientific, Cat# M7514) at 37 °C overnight, respectively. The next day, the cells were washed with PBS and gently scraped off the dish with a cell scraper. They were mixed in different ratios, and the buffer well in each of the four micro-runners was loaded with 120 µL culture media containing 2 × 10^4^ cells. The sample well in the four micro-runners was loaded with 30 µL human CD44 antibody–magnetic bead conjugates (Miltenyi Biotec). We then used two different rotating modes to label and sort the cells by the centrifugal device. The first rotating mode was to let the microfluids flow to the reaction area from the buffer and sample well. The purpose was to let the labeling cells react and combine with the human CD44 antibody–magnetic bead conjugates. Thence, the microfluids of the reaction area flowed to the reservoir area by the second rotating mode. At this time, the labeling cells were adsorbed at the capture well, and the non-labeling cells flowed to the reservoir area. The experimental results achieved the function of sorting between labeling and non-labeling cells.

**FIGURE 3:**
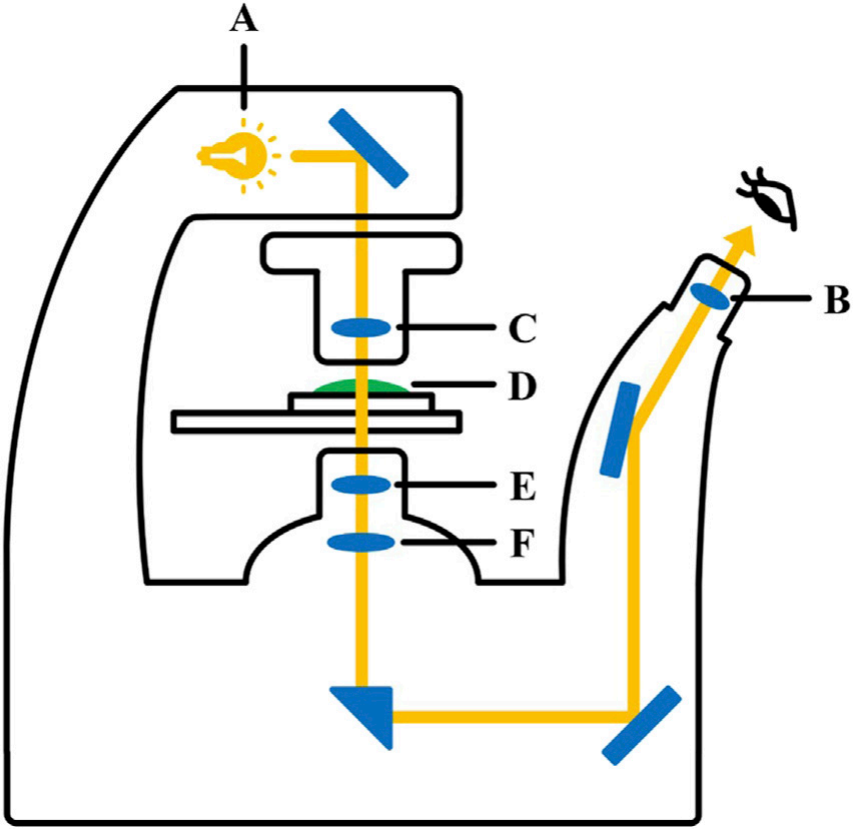
Inverted fluorescence microscopy. A. light source; B. condenser lens; C. eyepiece; D. observation sample; E. objective lens; F. imaging lens.

To image the cells, the LoaD was placed on the stage of a fluorescence microscope (Nexcope, NIB610) (Figure 3). The cells in the reservoir area were imaged first, and the fluid was immediately collected for subsequent flow cytometric analysis (BD Acuri C6 Plus, BD Science). The magnets were gently removed from the back of the LoaD before the cells retained at the magnetic area were imaged. The cells were also subjected to flow cytometric analysis to measure the cells retained at the magnetic area. For all flow cytometric experiments, cells collected from either the magnetic or reservoir areas were resuspended in 1 mL saline buffer, and the first 1000 cells were counted.

## 3 Results and discussion

### 3.1 Performance of the centrifugal device

**FIGURE 4:**
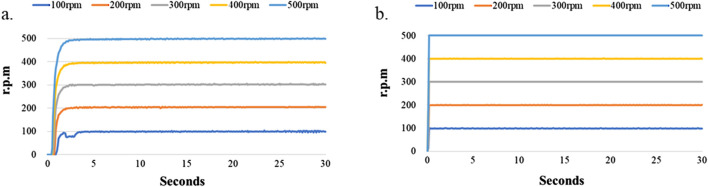
Stability test of the LoaD at higher rotational speed. The LoaD was mounted onto a motor shaft driven by either **(a)** a DC motor or **(b)** a servomotor at a designated speed for 30 s.

Since the LoaD in the present study relied on a combination of microchannel geometry and centrifugation to separate cells within a biological sample, it is essential that the disk maintain its balance and spin at a constant speed over some time without falling off the motor shaft. In addition, the lower cost and higher performance of the centrifugal device are our novel target. Therefore, we tested the rotating performance of the DC motor and servo motor devices (Figures 1a, b). Figure 4 shows the experimental results. Figure 4a shows the relationship of the rotating speed and time when the DC motor is driving at 100, 200, 300, 400, and 500 rpm and 30 s. We found the difference of the maximum and minimum speeds to be 7 rpm when the rotating speed of the DC motor device was set at 100 rpm. The difference of the maximum and minimum speeds was 6 rpm when the rotating speed of the DC motor device was 200 rpm. The difference of the maximum and minimum speeds was 9 rpm when the rotating speed of the DC motor device was 300 rpm. The difference between the maximum and minimum speeds was 8 rpm when the rotating speed of the DC motor device was 400 rpm. The difference of the maximum and minimum speeds was 6 rpm when the rotating speed of the DC motor device was 500 rpm. However, the difference of the maximum and minimum speeds could be controlled to within 1 rpm when the rotating speed of the servo motor device was between 100 and 500 rpm. The experimental results show that the servo motor device performs better than the DC motor device at the same rotating speed . According to Branderhorst (2024), the servomotor has the higher control performance, but its cost is higher than the DC motor.

**FIGURE 5:**
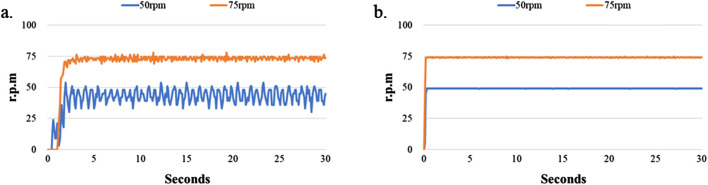
Stability test of LoaD at lower rotational speed. The LoaD was allowed to rotate on a motor shaft driven by either **(a)** a DC motor or **(b)** a servomotor at a designated speed for 30 s.

Figure 4a shows that the DC motor system exhibits poor stability when the motor speed was 100 rpm. Therefore, we tested the performance of the motor system when the rotating speed was 50 and 75 rpm. The experimental results are shown in Figure 5. We found the difference to be about 9 rpm. when the rotating speed of the DC motor was 75 rpm. However, the difference of the servo motor was only 2 rpm. under the same rotating speed. The difference increased to 24 rpm when the DC motor’s rotating speed slowed to 50 rpm, and the difference of the servo motor was 16 rpm at the same rotating speed. We found that although the servo motor had better performance than the DC motor when the rotating speed was set below 100 rpm, the rotating speed stability of the servo motor became worse when the motor speed decreased. This is due to the relationship between the specifications of the motor torque and encoder pulses (Lu et al., 2018; Othman et al., 2018).

### 3.2 Performance of microfluidic flow in the LoaD pattern

The geometric design of the microfluidic pattern will affect the performance of the cell sorting (Glass et al., 2012). In addition, Pishbin et al. (2020) showed that the rotation speed of the LoaD also affects sorting performance. Therefore, different rotating speeds and times may have different sorting performances under the same geometry design of the microfluidic pattern. Thus, we investigated the fluid flow behavior of microfluidics within 120 µL in the runner of the LoaD based on Figure 2 using the servomotor device. The experimental process also tested the optimal angle design of the LoaD’s mixing runner θ.

**FIGURE 6:**
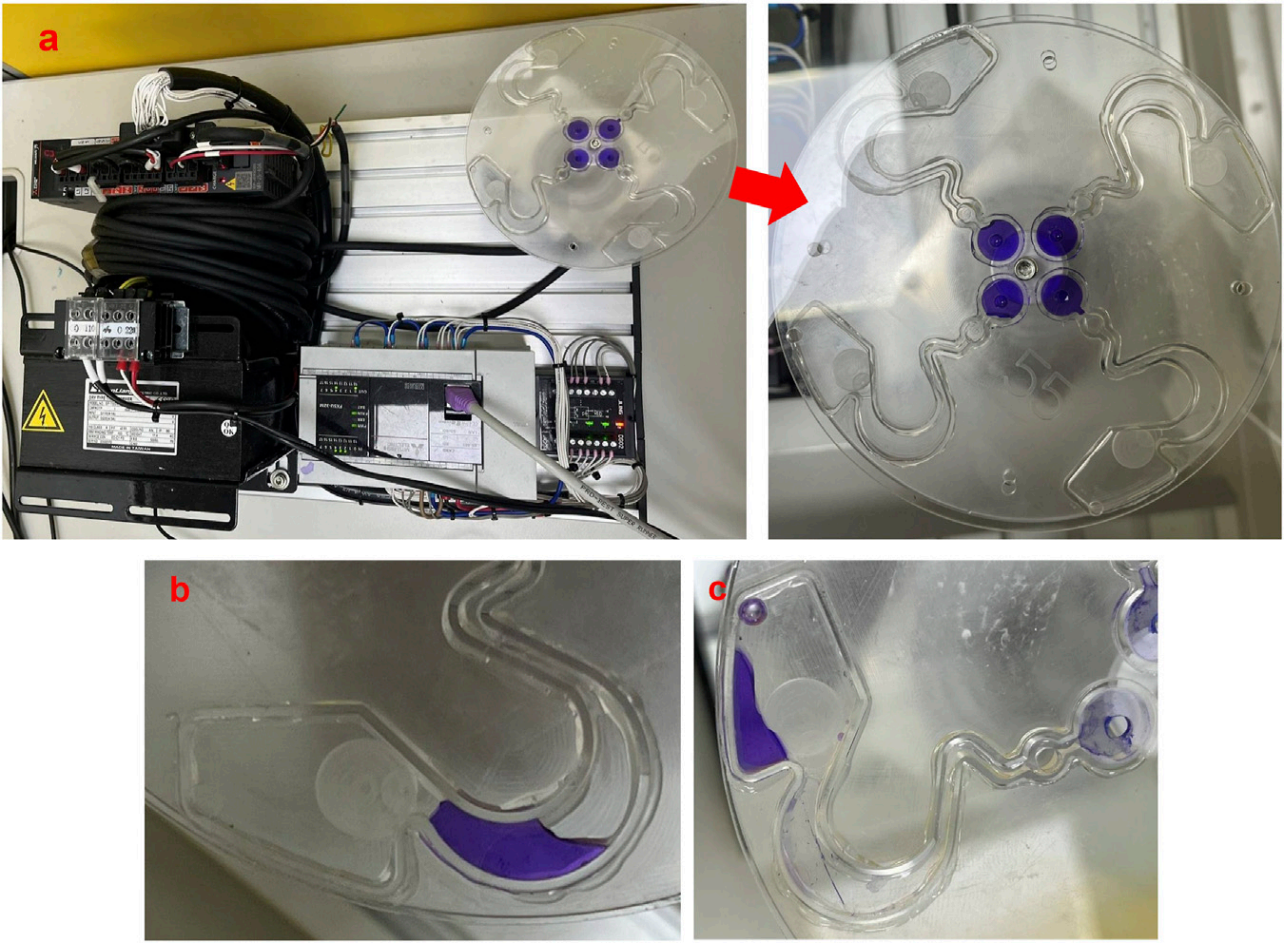
Performance of microfluidic flow in the pattern of the LoaD. **(a)** Coomassie Brilliant Blue R-250 of microfluids in the buffer well. **(b)** Microfluid flow to the reaction area from the buffer well by the first rotating mode of the centrifugal device. **(c)** Microfluid flow to the reservoir area from the reaction area by the second rotating mode of the centrifugal device.

Figure 6a shows 120 µL Coomassie Brilliant Blue R-250 of microfluidics poured into the buffer well. We then drove the centrifugal device of the servomotor with 200∼250 rpm for 30∼50 min (Lin Y. T. et al., 2021). We tested difference angle of the mixing runner θ at 45°, 50°, 55°, 60°, 65°, 70°, 75°, 80°, 85°, and 90°. Each angle of the mixing runner was tested ten times. The results demonstrated that the microfluids of the buffer well are able to flow to the reaction area passing to the sample well and mixing runner (Figure 6b). We call this the first rotating mode of the centrifugal device. We again drove the centrifugal device of the servo motor at 200∼250 rpm and 20∼25 min. This was the second rotating mode of the centrifugal device. We found that the fluid of the reaction area is able to flow to the reservoir area, passing to the capture well in the second rotating mode of the centrifugal device (Figure 6c). The experimental results verify that the novel centrifugal device of the servo motor can control the fluid flow process with the same geometric pattern design of the LoaD by different rotating modes. The optimal rotating modes of the centrifugal device are in Table 2, and the optimal angle of the mixing runner design was within the range of 55°∼65°.

**TABLE 2:** Optimal rotating modes of the centrifugal device.

	Rotating speed	Rotating time
First rotating mode of the centrifugal device	200 r.p.m	15∼20 min
Second rotating mode of the centrifugal device	250 r.p.m	20∼25 min

### 3.3 Purification of cancer cells from biological mixtures

To demonstrate that our novel centrifugal device has the potential for clinical application, the purification efficiency of the disk was examined as in Figure 6. The buffer area was loaded with antibody–magnetic-bead conjugates recognized specifically for CD44 (CD44 microbeads) in the buffer area. CD44 is a cell receptor known for its role in cancer progression and is a diagnostic marker for early tumor detection (Yaghobi et al., 2021). The sample area was loaded with culture media that contained human breast cancer cells MDA-MB-231 and human non-cancer cells HEK293. CD44 receptors are found abundantly on MDA-MB-231 cell surfaces but not on HEK293 (Kim et al., 2021; Dinthe, 2023).

The MDA-MB-231 cells were pre-stained with Mitotracker Red, whereas HEK293 cells were pre-stained with Mitotracker Green. The two cell types were mixed in culture media and subjected to cell separation in the LoaD (Figure 6a). After being centrifuged counterclockwise for 20 min, the fluid in the buffer and sample areas were mixed at the reaction area (Figure 6b), with no trace of liquid in other compartments. The LoaD was removed from the motor shaft and then placed in a 4 °C incubator for 1 hour, allowing the CD44 microbeads to react with the MDA-MB-231 cancer cells. Afterward, the LoaD was centrifuged at 250 rpm for 25 minutes, and it was noticed that all the liquid was retained in the reservoir area with no trace of fluid in the other compartment (Figure 6c). It was expected that the microbead-bound MDA-MB-231 cells would be retained at the capture well (magnetic area) (Figure 2) while the rest of the biological components would end up at the reservoir area.

**FIGURE 7:**
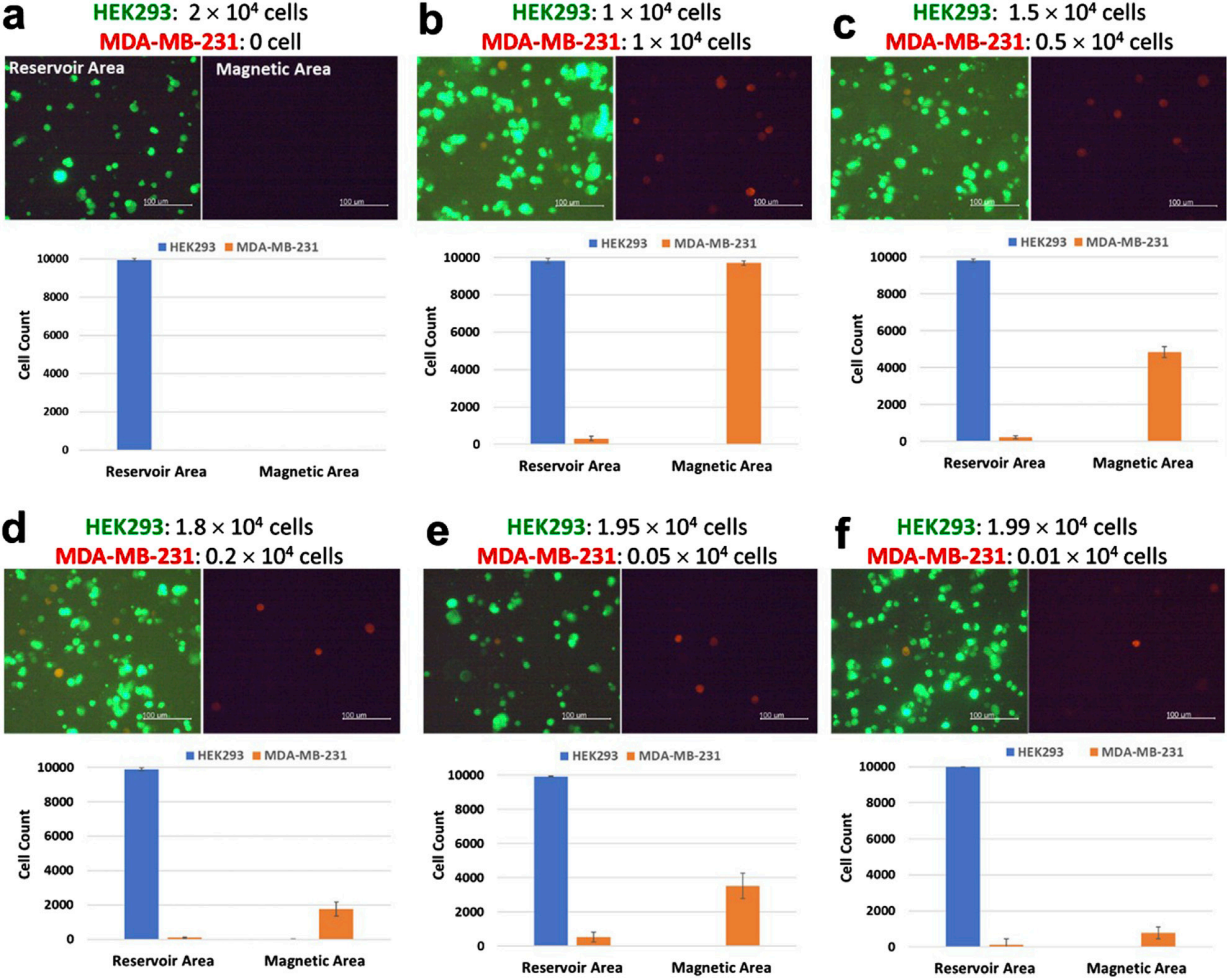
Purification of human breast cancer cells MDA-MB-231 from a cell mixture. The day before the purification experiment, HEK293 cells were stained with Mitotracker Green, and MDA-MB-231 cells were stained with Mitotracker Red. **(a–f)** A total of 2 × 10^4^ cells that consisted of specified ratios of HEK293 cells and MDA-MB-231 cells were mixed in the same cell culture media at the final volume 120 µL. After the centrifugation steps, fluorescence images of the cells retained at either the reservoir area or the capture well were taken (scale bar, 100 µm). Subsequently, cells were removed and subjected to flow cytometric analysis. The number of Mitotracker Green stained HEK293 cells (green) and Mitotracker Red stained MDA-MB-231 cells (red) detected in either the reservoir area or the capture well (N = 4).

Figure 7 shows that the purification efficiency of the LoaD was examined with culture media containing different ratios of MDA-MB-231 to HEK293 cells. In the absence of any MDA-MB-231 cells, no cells were detected in the capture well either by fluorescence imaging or flow cytometric analysis, indicating the CD44 antibody–magnetic-bead conjugates were specific for cells that expressed CD44 (Figure 7a). When exposed to the conditioned media containing 1 × 10^4^ of MDA-MB-231—which represented 50% of the total cell population in the cell culture media—the LoaD managed to capture >99% of the cancer cells in that sample (Figure 7b). Moreover, the number of non-targeted HEK293 cells detected at the magnetic area was extremely low, as revealed by flow cytometric analysis. Likewise, in the culture media that had 0.5 × 10^4^, 0.2 × 10^4^, or 0.05 × 10^4^ MDA-MB-231 cells (equivalent to 25%, 1%, and 0%, 2.5% of the total cell populations, respectively), approximately 85% or greater of the cancer cells were retained at the magnetic area (Figures 7c–e). Additionally, the non-cancerous HEK293 cells were only detected at the reservoir area but not at the magnetic area, indicating that the LoaD has high purification capability.

Studies examining cell separations via magnetic microbeads and centrifugal microfluids have indicated that apart from microchannel geometry, the magnetic strength of magnets also affected separation efficiency (Ma et al., 2019). Assuming that all MDA-MB-231 cells had CD44 microbeads bound to their surfaces, it was suspected that the strength of the magnets installed beneath the magnetic area was not strong enough to capture all CD44 microbead-bound cells. A possible solution would be to increase the area of the magnet (Fu et al., 2016), thus increasing the chance of capturing and retaining the CD44 microbead-bound cells while the LoaD is being centrifuged. Alternatively, instead of increasing the size of the magnets, the magnetic strength could be increased by installing stacked magnets at the back of each four microchannels (Liu et al., 2022). Similarly, increasing the concentration of the CD44 microbeads could also improve the capture efficiency of the microbead-bound cells (Li et al., 2022). Nevertheless, the LoaD in the present study could still retain ∼85%–∼99% of the MDA-MB-231 cells at the capture well while being centrifuged at 250 rpm for 25 minutes. This indicated that the microchannel geometry in the LoaD with the Pattern II design can effectively purify cancer cells from a biological sample.

Large numbers of the non-target cells were also likely to play a role in inhibiting the interactions between the magnets and the CD44 microbead-bound cells. The lowest number of MDA-MB-231 cells that the LoaD exposed to in the present study was 0.01 × 10^4^ MDA-MB-231 cells—equivalent to 0.5% of the total cell number (Figure 7f). Even at such a low number, the LoaD was able to capture ∼75% of the MDA-MB-231 cells, as demonstrated by the flow cytometric data. This indicated that a high concentration of non-target cells could prevent the magnet from capturing the CD44 microbeads bound cells as the sample was spun from the mixing to the reservoir area.

Clinically, the average concentration of circulating tumor cells in a cancer patient’s blood is only one to ten cells per mL (Lin D. et al., 2021), whereas 1 mL of blood contains a few million white blood cells and a billion red blood cells (Yamamoto et al., 2020). Some studies have indicated that diluting biological samples before subjecting them to a diagnostic kit enhances the chance of targeted cells being detected (Galanzha et al., 2019; Endesfelder, 2019). Accordingly, a serial dilution on a biological sample would be needed to justify our LoaD, which could be used as a diagnostic tool for detecting an extremely low number of cells, such as circulating tumor cells in human blood.

Ideally, for any given cell separation technology, it would be best to separate and purify a specific biological target in a label-free manner. Although there have been some promising progress in the development of LoaD to enable the label-free isolation of target cells, most of the reported studies still rely on separation based on size (Zhu et al., 2020b). It had been demonstrated that LoaDs separating cells based on size had a higher chance of contaminating the targeted cells with unwanted cells than those utilizing an antibody-labeling method (Sunkara et al., 2021). Furthermore, the CD44 microbeads bound on the surfaces of MDA-MB-231 could be easily removed from the cell surfaces with a well-defined microbeads removal reagent. Such a reagent does not harm the cells, enabling them to be subjected to further analysis (Seitz et al., 2021).

## 4 Conclusion

According to the special pattern design of the LoaD, the study created a novel centrifugal device to control microfluidic flow, stagnation, and reflow in the runner of the pattern by different rotating modes of the motor. It also showed that the servomotor can steadily control the rotating speed in the novel centrifugal device of the LoaD compared to the DC motor. In addition, the device installed magnets on the back of the LoaD’s capture well to adsorb the target cells. The target cells are bound with micro magnetic beads. Therefore, the study and the novel centrifugal device successfully isolated human breast cancer cells from a cell culture medium containing non-cancerous cells. The sorting process was steady, and the separated cancer cell solution has been verified to be free of non-cancerous cells. The study also verified that the novel detection system has the application development advantages of simply operation, faster detection, and lower cost of the centrifugal device. In addition, the LoaD can be easily mass-produced by injection molding to achieve disposability and low cost—the main demands for the commercialization of medical products. Our novel device can thus be used as a point-of-care testing (POCT) device for applications in clinics. In addition, we found that the strength of the magnetic field and the concentration of CD44 micro beads affect the ability of the cancer cells captured. Therefore, we will improve its ability to capture cancer cells by optimizing the strength of the magnetic field and adjusting the CD44 micro-bead concentrations.

## Data Availability

The original contributions presented in the study are included in the article/Supplementary Material; further inquiries can be directed to the corresponding author.

## Glossary

a: Acceleration of the microfluidics
B: Magnetic field intensity
**CFD**: Computational fluid dynamics
**CNC**: Computer numerical control
d: Diameter of the runner in the LoaD
**DC**: Direct current
De: Dean flow
**EtOH**: Ethyl alcohol
$f_{c}$: Inertial force
$f_{m}$: Magnetic force
L: Characteristic length of the rectangle
**LoaD**: Lab-on-a-disc
m: Qquality of the particle
P: Pressure
**POCT**: Point-of-care testing
R: Runner radius
Re: Reynolds number
t: Time
V: Volume of magnetic particle
∆x: Effective magnetic volumetric susceptibility
τ: Shear stress
u: Velocity of fluid
μ: Viscosity coefficient
$\mu_{0}$: Magnetic permeability of vacuum
δx: Fluid element in the x space
δy: Fluid element in the y space
δz: Fluid element in the z space
y: Unit element of fluid in the LoaD runner
ρ: Fluid density
$\rho_{0}$: Density of incompressible flow
ω: Angular velocity
θ: Angle
∆θ: Angle of movement
