# Supplementary material for: Enhanced cancer cell sorting using lab-on-a-disk pattern design with magnetic and centrifugal forces
Source: Front Bioeng Biotechnol. 2025 Aug 1;13:1611313. doi: 10.3389/fbioe.2025.1611313 (PMC12354510; doi:10.3389/fbioe.2025.1611313)
Supplement: Supplementary file 1 [file Supplementaryfile1.docx]

Supplementary Material

# Table 1 The boundary condition set for the simulation for the simulation.

| The volume of buffer liquid | The volume of sample liquid | The rotating speed of the platform |
| --- | --- | --- |
| 150 µL | 40 µL | First rotating mode 100 r.p.m. / 10 minutes.  Second rotating mode 250 r.p.m. / 10 minutes. |

| 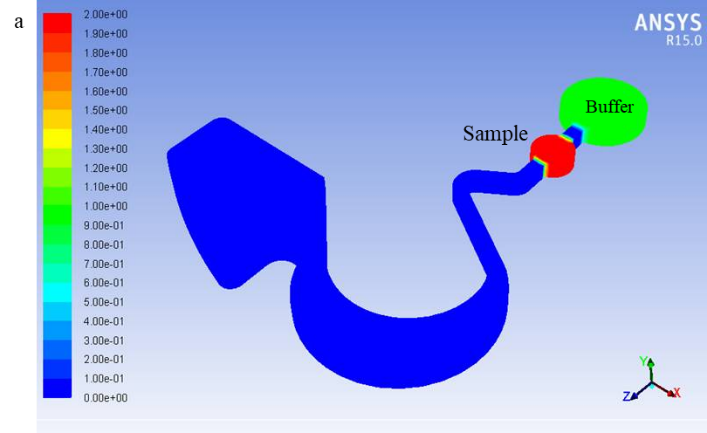 | 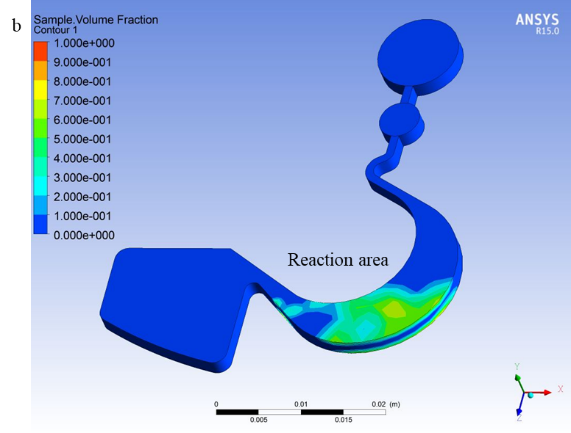 |
| --- | --- |
| 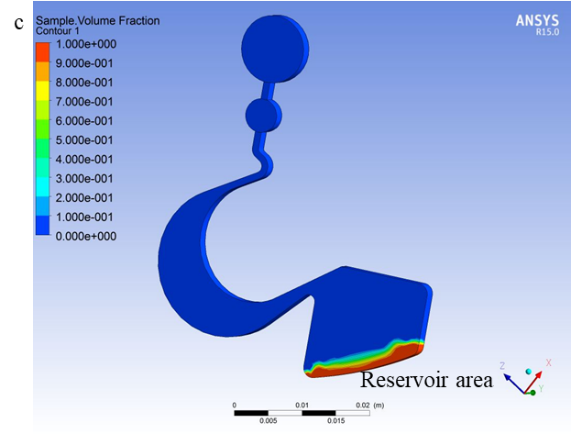 | 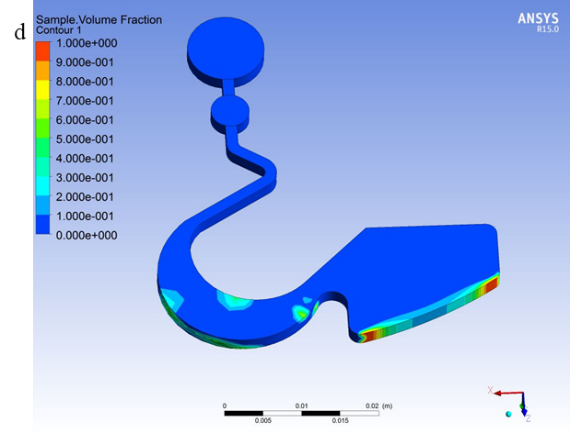 |

**Supplementary Figure 1.** The pattern design of the LoaD is done using CAE simulation. The centrifugal rotating mode is divided into two modes. The first rotating mode is that the liquid of the sample well flows to the reaction area and does not flow to the reservoir area, as shown in (a) and (b). The second rotating mode is that the liquid of the reaction area flows to the reservoir area, as shown in (c). (d) the overflow of the sample liquid is shown when the first rotating mode is over 200 r.p.m.

| 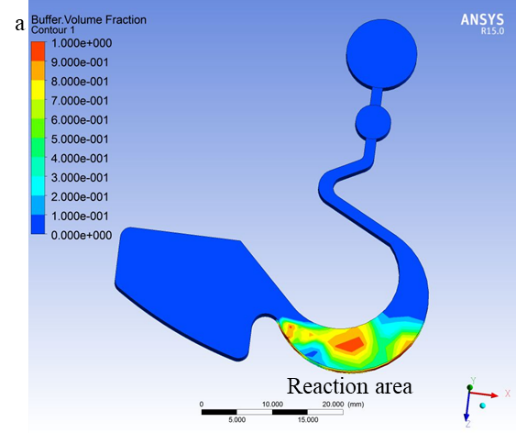 | 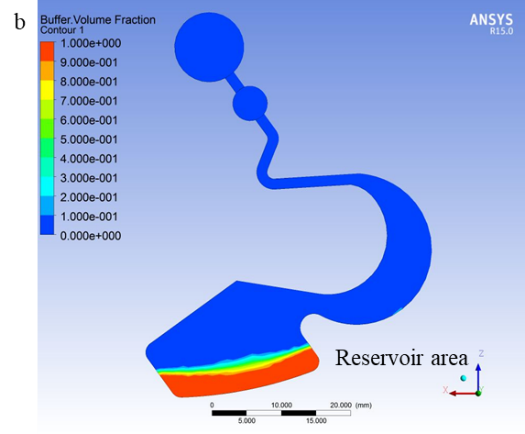 |
| --- | --- |
| 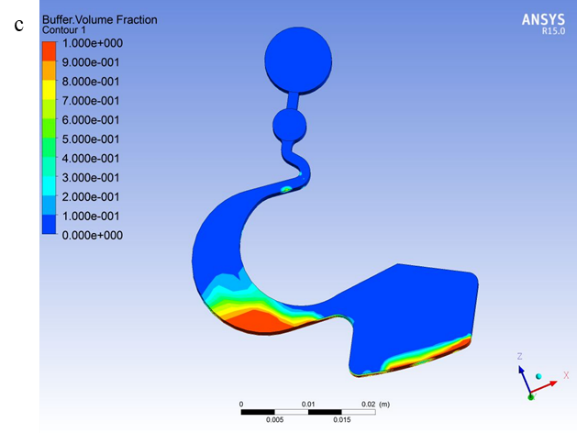 |  |

**Supplementary Figure 2.** The pattern design of the LoaD is done using CAE simulation. The centrifugal rotating mode is divided into two modes. The first rotating mode is that the liquid of the buffer well flows to the reaction area and does not flow to the reservoir area, as shown in (a). The second rotating mode is that the liquid of the reaction area flows to the reservoir area, as shown in (b). (c) the overflow of the buffer liquid is shown when the first rotating mode is over 200 r.p.m.

| 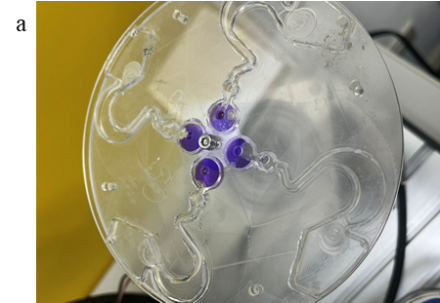 | 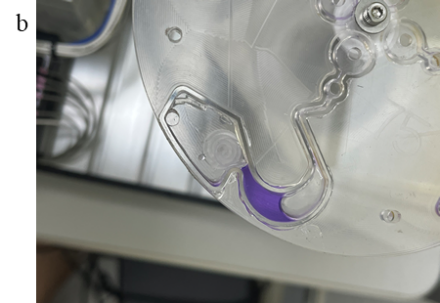 |
| --- | --- |
| 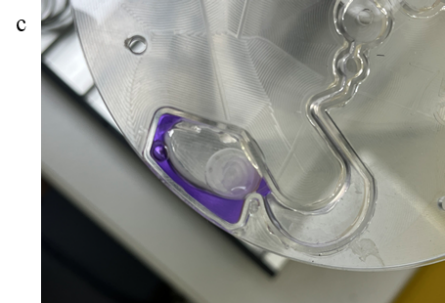 | 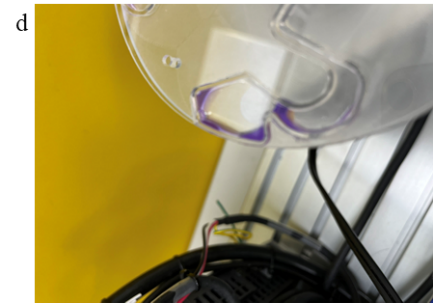 |

**Supplementary Figure 3.** The microfluidic flow in the pattern of the LoaD by centrifugal device drive. (a) is the microfluidics put into the buffer well. (b) is the microfluidics flow into the reaction area from the buffer well under the first rotating mode of the centrifugal device. (c) is the microfluidics flow into the reservoir area from the reaction area under the second rotating mode of the centrifugal device. (d) is the phenomenon of overflow when the LoaD rotates at the first rotating mode.
